# The United States Revised Uniform Anatomical Gift Act (2006): New challenges to balancing patient rights and physician responsibilities

**DOI:** 10.1186/1747-5341-2-19

**Published:** 2007-09-12

**Authors:** Joseph L Verheijde, Mohamed Y Rady, Joan L McGregor

**Affiliations:** 1 Departments of Physical Medicine and Rehabilitation, Mayo Clinic Hospital, Mayo Clinic Arizona, 5777 East Mayo Boulevard, Phoenix, Arizona, 85054, USA; 2 Critical Care Medicine, Mayo Clinic Hospital, Mayo Clinic Arizona, 5777 East Mayo Boulevard, Phoenix, Arizona, 85054, USA; 3 Department of Philosophy, Arizona State University, 300 East University Drive, Tempe, Arizona, 85287, USA

## Abstract

Advance health care directives and informed consent remain the cornerstones of patients' right to self-determination regarding medical care and preferences at the end-of-life. However, the effectiveness and clinical applicability of advance health care directives to decision-making on the use of life support systems at the end-of-life is questionable. The Uniform Anatomical Gift Act (UAGA) has been revised in 2006 to permit the use of life support systems at or near death for the purpose of maximizing procurement opportunities of organs medically suitable for transplantation. Some states have enacted the Revised UAGA (2006) and a few of those have included amendments while attempting to preserve the uniformity of the revised Act. Other states have introduced the Revised UAGA (2006) for legislation and remaining states are likely to follow soon.

The Revised UAGA (2006) poses challenges to the Patient Self Determination Act (PSDA) embodied in advance health care directives and individual expression about the use of life support systems at the end-of-life. The challenges are predicated on the UAGA revising the default choice to *presumption of donation intent *and the use of life support systems to ensure medical suitability of organs for transplantation. The default choice trumps the expressed intent in an individual's advance health care directive to withhold and/or withdraw life support systems at the end-of-life. The Revised UAGA (2006) overrides advance directives on utilitarian grounds, which is a serious ethical challenge to society. The subtle progression of the Revised UAGA (2006) towards the presumption about how to dispose of one's organs at death can pave the way for an affirmative "duty to donate". There are at least two steps required to resolve these challenges. First, physicians and hospitals must fulfill their responsibilities to educate patients on the new legislations and document their preferences about the use of life support systems for organ donation at the end-of-life. Second, a broad based societal discussion must be initiated to decide if the Revised UAGA (2006) infringes on the PSDA and the individual's right of autonomy. The discussion should also address other ethical concerns raised by the Revised UAGA (2006), including the moral stance on 1) the interpretation of the refusal of life support systems as not applicable to organ donation and 2) the disregarding of the diversity of cultural beliefs about end-of-life in a pluralistic society.

## Background

In 1990, the U.S. Congress passed the Patient Self-Determination Act (PSDA) reinforcing individuals' rights to determine their final health care. The PSDA became effective in 1991 so that patients can make decisions about their medical care and specify whether they want to accept or refuse specific medical care [1]. Patients' wishes can be clearly documented at an earlier point of time in advance health care directives and/or patients can identify legally authorized representatives to make health care decisions (power-of-attorney for health care) on their behalf in the event of an incapacitating illness.

The PSDA requires Medicare and Medicaid providers, including hospitals, to give adult individuals, at the time of inpatient admission, certain information about their rights under state laws governing advance health care directives, including: (1) the right to participate in and direct their own health care decisions; (2) the right to accept or refuse medical or surgical treatment; (3) the right to prepare advance health care directives and (4) information on the provider's policies governing the utilization of these rights [2].

## Scope of advance health care directives

Almost 16 years later, advance health care directives and power-of-attorney for health care still play a limited, yet important, role in assisting with health care decisions about the use of life support systems and medical technologies at the end-of-life [3]. About 21% of critically ill patients admitted to intensive care units for life support systems at the end-of-life have formal advance health care directives [4].

Criticisms have been rightfully expressed concerning the current deficiencies of construction, documentation, accessibility and applicability of advance health care directives [5]. The main reasons limiting the applicability of advance health care directives include: 1) physicians' uncertainties about diagnosis, treatment efficacy, and prognosis and 2) lack of knowledge, insight, and courage of persons authorized as power-of-attorney for health care to fulfill their assigned roles. These shortfalls raise concerns about the effectiveness of advance health care directives to prepare patients and families for uncertain and difficult decisions at the end-of-life [6]. To accommodate these concerns, advance care planning should be built on effective communication to individualize medical care and decision making despite future uncertainties. Advance care planning requires physicians to take time to discuss advance health care directives with patients and identify the specific circumstances in which care preferences should be followed [5].

Considering the above shortfalls, this commentary highlights additional and unique challenges posed by the Revised Uniform Anatomical Gift Act (UAGA) 2006 on advance health care planning and directives about the use of life support systems at the end-of-life [7]. Some states have already enacted the Revised UAGA (2006) and a few of those have included amendments while attempting to preserve the uniformity of the revised Act [8]. Other states have introduced the Revised UAGA (2006) for legislation and remaining states are likely to follow soon.

## Scope of deceased organ donation

In 2006, the publication of two influential reports from the Institute of Medicine and National Conference on Donation After Cardiac Death opened a new era for deceased organ donation [9,10]. Traditionally, organs for transplantation have been donated by individuals who fulfilled strict criteria of neurologic or brain death and had already been on life support systems [11]. Organ donation after cardiac death applies to individuals who sustain death because of circulatory or cardiorespiratory arrest and without the requirement for antecedent neurologic or brain death criteria. The two reports conclude that donation after cardiac death is an ethically acceptable form of organ donation. As of January 2007, federal regulations require Medicare-approved hospitals to design policies and procedures for donation after cardiac death from patients at or near death [12].

## Scope of the Revised UAGA (2006)

The National Conference of Commissioners on Uniform State Laws (NCCUSL) promulgated the Revised UAGA (2006) with the substantial and active participation of the major stakeholders representing donors, recipients, physicians, procurement organizations, regulatory agencies and the US Department of Health & Human Services. The stakeholders represented a broad spectrum of organizations with special interest or advocacy for the practice of organ transplantation. The primary intent of revising the UAGA in 2006 was to solve the critical organ shortage by maximizing the likelihood of organ donation. To accomplish this objective, the Revised UAGA (2006) increases opportunities of organ procurement after cardiac death for transplantation [7]. The anatomical gifting of organs (heart, lungs, kidneys, liver, pancreases, small bowel, etc.) after cardiac death requires the initiation and/or continuation of life support systems at the end-of-life to ensure their medical suitability for transplantation.

The Revised UAGA (2006) reaffirms that if a donor has a document of gift, there is no reason to seek consent from the donor's family as they have no right to give it legally [7]. If an individual has not made a document of gift during life, the Revised UAGA (2006) presumes the intent to donate organs and, therefore, has expanded the list of persons (in section 9a) who can consent to organ donation on behalf of that individual. The Revised UAGA (2006) considers that every individual has the right to donate his (her) organs at or near death. Finally, if an individual prefers not to donate, this must be documented in a signed, explicit refusal.

The Revised UAGA (2006) section 14 was drafted in accordance with the controlling federal law requiring hospitals to notify an organ procurement organization (OPO) of any individual whose death is imminent or who has died in-hospital to increase opportunities of organ procurement for transplantation [13]. In cases of potential organ donation, measures necessary to ensure the medical suitability of an organ for transplantation are administered to a patient who is dead or near death to allow time for determination if the patient could be a prospective donor. That provision applies to situations of sudden in-hospital or out-of-hospital cardiac death when resuscitation is unsuccessful [9]. Organ preservation requires the administration of life support systems until the OPO has determined if a patient can be a prospective donor. The Revised UAGA (2006), section 14(c), requires life support systems already administered to a patient who has been referred to the OPO for evaluation to be continued until it is determined that the patient has organs that are medically suitable for transplantation. This section applies to a patient who is already on life support systems either in the emergency department or intensive care unit at the end-of-life.

The Revised UAGA (2006), section 14, emphasizes the general direction in an advance health care directive or power-of-attorney for health care that the patient's wish *not to have life prolonged *by the administration of life support systems should *not *be construed as an expression of a contrary intent for the use of life support systems for donation purpose [7]. In effect, a patient on life support systems at the end-of-life (and without signed refusal) is defaulted to the presumption of intent to donate organs, and therefore life support systems cannot be withdrawn until the OPO's evaluation for organ donation is complete. The OPO can then determine that the patient *is *a prospective donor.

If determined to be a prospective donor, the Revised UAGA (2006), section 21, creates a default rule requiring that measures necessary to ensure the medical suitability of an organ for transplantation may not be withheld or withdrawn. The initiation and/or continuation of life support systems is the default rule and overrides a prospective donor's expression in an advance health care directive not to have life prolonged by life support systems. To resolve the tension between the presumed intent to donate organs and the expressed intent not to have life support systems administered merely to prolong life, section 21 presumes that for a prospective donor the desire to save lives by making an anatomical gift trumps the desire to have life support systems withheld or withdrawn. The Revised UAGA (2006) requires a prospective donor to expressly provide *contrary intent *to prevent the use of life support systems for organ donation purposes in either a declaration or advance health care directives.

In 2007, an amendment was introduced to the Revised UAGA (2006), section 21, to recognize the conflict between initiation and/or continuation of life support systems based on becoming a prospective donor and the expressed wishes of appropriate end-of-life care in advance health care directives. Section 21(b) (2007) requires the attending physician to resolve the conflict with the prospective donor or surrogate decision maker for clarification of intent and any contraindications for appropriate end-of-life care.

## The Revised UAGA (2006) and advance health care directives

**Figure 1:**
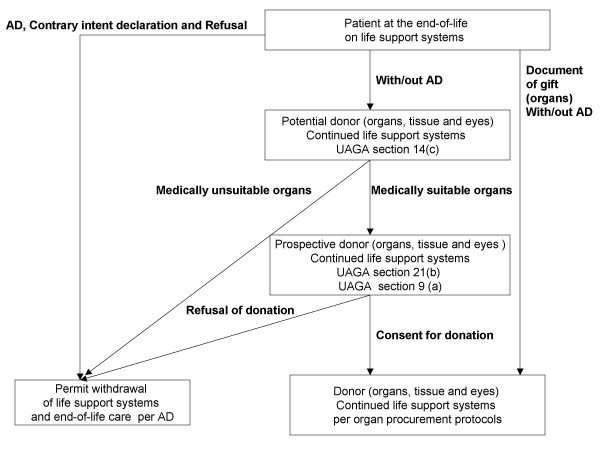
**The revised Uniform Anatomical Gift Act (UAGA) 2006, advance health care directives (AD) and use of life support systems at the end-of-life**. The UAGA (2006) Section 14(c) defaults a patient already on life support systems to the presumption of intent for organ donation (i.e. potential donor) and mandatory notification of organ procurement organization for evaluation. Life support systems cannot be withdrawn in a potential donor until organ procurement organization has completed the evaluation of medical suitability of organs for transplantation. If the organ procurement organization has determined that a potential donor has organs medically suitable for transplantation, the potential donor becomes a prospective donor. For a prospective donor, life support systems cannot be withheld or withdrawn. For a prospective donor, section 21(b) requires the attending physician to resolve the conflict between intent in advance health care directives to withhold and/or withdraw life support systems at the end-of-life verus their use for organ donation purpose. Section 9(a) expands the list of persons who can consult, on behalf of a prospective donor, with the attending physician to resolve the aforementioned conflict and provide donation consent (or refusal). Document of gift or donation consent permits the use of life support systems and organ procurement protocols on donors. If a potential donor has medically unsuitable organs, refusal of gift or contrary intent declaration to instruct the withholding and/or withdrawing of life support systems for organ donation purpose, life support systems can be withdrawn and end-of-life care is provided as expressed in advance health care directives.

With the new UAGA legislation, advance health care planning should include education on the new requirement of the Revised UAGA (2006) about the use of life support systems for organ donation at the end-of-life. These changes are predicated on the UAGA revising the default choice to presumption of donation intent and, therefore, the requirement for life support systems to ensure medical suitability of organs for transplantation. Figure 1 summarizes how document of gift, advance health care directives, contrary intent declaration and refusal determine the pathway for withholding and/or withdrawal of life support systems at the end-of-life. Only a refusal and contrary intent declaration can permit the withholding and/or withdrawal of life support systems and the administration of appropriate end-of-life care as expressed in advance health care directives (figure 1).

Patients with documents of gift are considered donors irrespective of advance health care directives and they are required to comply with organ procurement protocols (figure 1). In the default pathway, (i.e. the absence of refusal and contrary intent declaration) life support systems are required, irrespective of advance health care directives, until the evaluation of medical suitability of organs for transplantation has been completed. Regardless of whether it is morally right to construe refusal of life support in an advance directive as not applicable for organ donation, the final authority of the OPO to determine donor's medical suitability raises additional normative ethical issues. Three factors can inflate the pool of prospective donors unpredictably: 1) the OPO can apply liberal criteria about medical suitability for donation because the definition of eligible donors is very broad [12], 2) the OPO has the discretion to offer for transplantation organs of marginal (inferior) quality that would be otherwise rejected [14], 3) the OPO's decisions and actions are defaulted to be "in good faith" and are the subject of immunity from criminal, civil and administrative liabilities [7]. Specific conditions such as overwhelming infections, disseminated malignancy and communicable infectious diseases are absolute exclusion criteria for organ donation. However, the majority of potential organ donors are unlikely to meet any of these specific exclusion criteria [15].

The laxity of criteria of medical suitability for donation is most disturbing to patients who become prospective donors without documents of gift and who have unequivocal advance health care directives expressing intent for withholding and/or withdrawal of life support systems at the end-of-life (figure 1). Under such circumstances, the Revised UAGA (2006) requires the attending physician to address and resolve the conflict between the use of life support systems for donation purposes and appropriate end-of-life care with families and/or surrogate-decision makers while keeping the patient on life support systems.

## The Revised UAGA (2006) and end-of-life care

In the endeavor to solve the critical organ shortage, the Revised UAGA (2006) has transformed the traditional 'altruistic' to a disturbing 'utilitarian' approach towards organ procurement for transplantation. National palliative and hospice care organizations [16,17] promoting excellence in end-of-life care should have been better represented as stakeholders when drafting the revised UAGA. As a consequence, the UAGA drafting committee was able to set aside the advancements in end-of-life care accomplished over the past decade [18,19]. While the drafting committee has refuted that the Revised UAGA (2006) was drafted to accomplish the goals of special interest groups [20], the Act undoubtedly has created unintended consequences jeopardizing the general public's interest and disregarding longstanding respect of individual autonomy and diversity of cultural beliefs about end-of-life in a pluralistic society. The premises underlying the subtle progression of the Revised UAGA (2006) towards the presumption about how to dispose of one's organs at or near death can pave the way for an affirmative "duty to donate" to the detriment of human liberty in a free society [21].

The Revised UAGA (2006) has not adopted presumed consent for organ procurement. Nevertheless, the most disturbing consequence of the Act is that patients will be forced to have life support systems initiated or continued while awaiting the assessment of their organs for donation. In an epidemiologic study of the use of intensive care at the end-of-life in the US, one in five Americans die using intensive care services [15]. It is likely that the Revised UAGA (2006) will further increase the ratio of Americans dying in intensive care units by legitimizing presumed consent for the use of life support systems for organ donation.

The application of life support systems for the purpose of organ donation without explicit consent is contraindicated at end-of-life and inconsistent with recommended practice guidelines for quality palliative care [19,22]. Life support systems have no palliative benefit and inflict unwarranted traumatic and distressing experiences to dying patients and their families [23,24]. While section 21(b) (2007) concedes to the obvious conflict between life support systems for organ donation and appropriate end-of-end life care for the dying patients, the amendment is insufficient to protect patients and families from potential violations of their rights to quality palliative care. Section 21(b) (2007) requires the attending physician to balance contraindications for end-of-life care against the need to preserve organs, which can only be done after the OPO has completed medical evaluation to determine if a patient can be considered a prospective donor. Section 21(b) (2007) also includes the OPO as an agent to assist with conflict resolution with regard to end-of-life care, yet, the same agent has other undisclosed incentives, i.e. maximizing organ procurement opportunities [12]. There are no real safeguards to prevent the OPO from either prolonging or manipulating end-of-life decision making for prospective donors in order to obtain donation consent.

The Revised UAGA (2006) requirement of life support systems for patients with clearly contrary end-of-life wishes represents a radical departure from the PSDA (1991) and original intent of advance health care directives. In fact, it can be argued that the Revised UAGA (2006) intrudes into patients' autonomy and infringes on their rights to self-determination of medical care at the end-of-life.

## Conclusion

**Figure 2:**
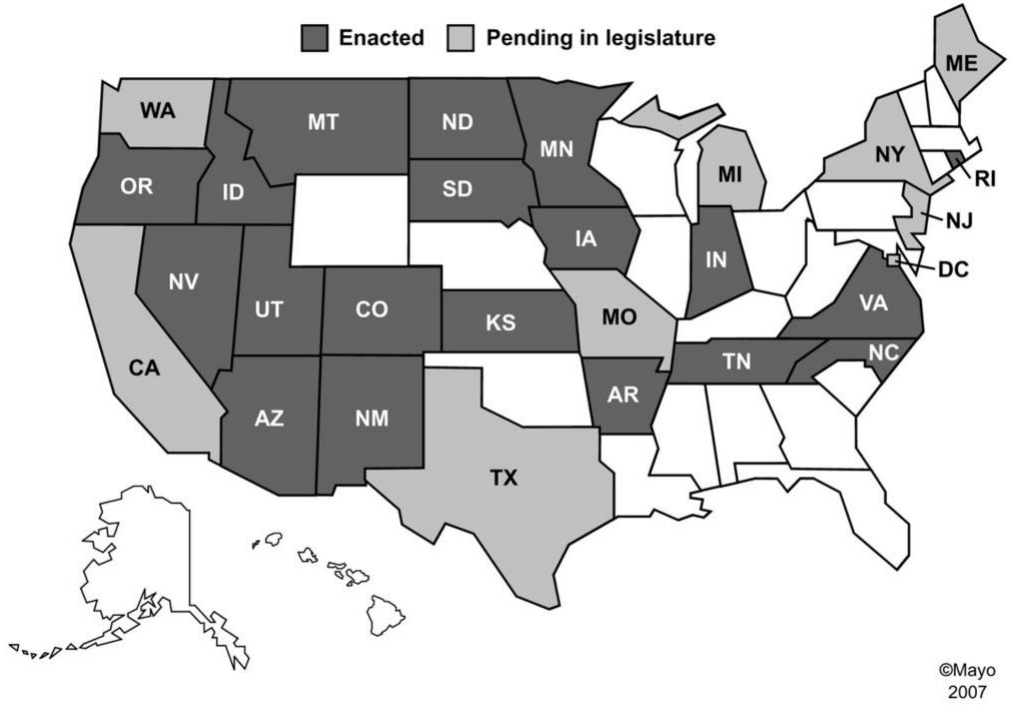
**The enactment status of the United States Revised Uniform Anatomical Gift Act (UAGA) 2006 as of September 2007.** The Revised Uniform Anatomical Gift Act (UAGA) 2006 is enacted in many states and a few of those have included amendments (dark shade areas). Other states have introduced the Revised UAGA (2006) for legislation (light shade areas). The data source is the National Conference of Commissioners on Uniform State Laws.

Some states have already enacted the Revised UAGA (2006) and a few of those have included amendments while attempting to preserve the uniformity of the revised Act (Figure 2). Other states have introduced the Revised UAGA (2006) for legislation and remaining states are likely to follow soon. The Revised UAGA (2006) increases physicians' and hospitals' responsibilities to fulfill their legal and moral obligations towards patients' rights for self-determination of their medical care and quality of palliation at the end-of-life. Therefore, it is imperative for patients, families and physicians to become familiar with the new US legislations about organ donation, so that the document of gift and advance health care directives are not in conflict and symbolize the commitment to patient's autonomous decision-making at the end-of-life. The premises underlying the subtle progression of the Revised UAGA (2006) towards the presumption about how to dispose of one's organs at or near death can pave the way for an affirmative "duty to donate" to the detriment of human liberty in a free society. Therefore, a broad based societal discussion must be initiated to decide if the Revised UAGA (2006) infringes on PSDA and the individual's right of autonomy. The discussion should also address other ethical concerns raised by the Revised UAGA (2006), including the moral stance on 1) the interpretation of the refusal of life support systems as not applicable to organ donation and 2) the disregarding of the diversity of cultural beliefs about end-of-life in a pluralistic society.

## Abbreviations

PSDA = Patient Self Determination Act

OPO = Organ procurement organization

UAGA = Uniform Anatomical Gift Act

US = United States

See table 1 for a glossary of terms.

**Table 1:** Glossary of terms

"**Advance health care directive**" means a power-of-attorney for health care or a record signed or authorized by a prospective donor containing the prospective donor's direction concerning a health care decision for the prospective donor.
"**Anatomical gift**" means a donation of all or part (an organ, an eye, or tissue) of a human body to take effect after the donor's death for the purpose of transplantation, therapy, research, or education. "
"**Contrary intent**" means a document expressing that no measures to be taken to ensure the medical suitability of an organ for transplantation and authorize and instruct the withholding and/or withdrawing of such medical measures and treatment including life support systems for that purpose.
"**Declaration**" means a record signed by a prospective donor specifying the circumstances under which life support systems may be withheld or withdrawn from the prospective donor.
"**Document of gift**" means a donor card or other record used to make an anatomical gift. The term includes a statement or symbol on a driver's license, identification card, or donor registry.
"**Donor**" means an individual whose body or part is the subject of an anatomical gift.
"**Health care decision**" means any decision regarding the health care of the prospective donor.
"**Life support systems**" means the use of machines and/or administration of medications for artificial support of vitals organs. Mechanical ventilators support the respiratory system. Medications and/or mechanical means (e.g. external cardiac compression devices, internal cardiac assist devices or artificial heart-lung machines) support the circulatory system.
"**Medically suitable organs**" means the determination of medical suitability of organs by the organ procurement organization who performs the examination and evaluation of potential donors.
"**Organ procurement organization**" means a private organization operating under government contract to provide services covering all aspects of deceased organ donation to include 1) donor evaluation, selection and consenting and 2) preparation, recovery and transportation of procured organs. Each organization is assigned to a specific geographic area or donation service area within the US. There are 58 organizations covering all states including the District of Columbia, Puerto Rico, the United States Virgin Islands, or any territory or insular possession subject to the jurisdiction of the US.
"**Organ procurement protocols**" means medical treatment and surgical procedures performed on donors to ensure successful procurement of viable organs for transplantation.
**"Power-of-attorney for health care" **means a legally authorized representative to make health care decisions on behalf of an individual in the event of an incapacitating illness and inability to make own health care decisions.
"**Prospective donor**" means an individual who is dead or near death and has been determined to have one or more parts that could be medically suitable for transplantation, therapy, research, or education. The term includes an individual who made an anatomical gift during life and, therefore, is a donor. The term also includes a non-donor individual at or near the time of death with parts that are medically suitable for donation who could become a donor if the individual's family made an anatomical gift (section 9). The term does not include an individual who made a refusal as the refusal bars other persons from making an anatomical gift on that individual's behalf.
"**Refusal**" means a record created that expressly states intent to bar other persons from making an anatomical gift of an individual's body or part.
"**Section 9 (a)**" sets a prioritized list of classes of persons (power-of-attorney for health care, spouse, adult children, parents, adult siblings, adult grandchildren, grandparents, an adult who exhibited special care and concern for the decedent, persons who were acting as the guardians of the person of the decedent at the time of death, any other person having the authority to dispose of the decedent's body) who can make an anatomical gift of a decedent's body or part if the decedent was neither a donor nor had signed a refusal. The same list of classes of persons can be consulted for section 21(b) whether they would be willing to make a gift when the prospective donor is near death.
"**Section 14(c)**" When a hospital refers an individual at or near death to a procurement organization, the organization may conduct any reasonable examination necessary to ensure the medical suitability of a part that is or could be the subject of an anatomical gift for transplantation, therapy, research, or education from a donor or a prospective donor. During the examination period, measures necessary to ensure the medical suitability of the part may not be withdrawn unless the hospital or procurement organization knows that the individual expressed a contrary intent."
"**Section 21(b)**" If a prospective donor has a declaration or advance health-care directive and the terms of the declaration or directive and the express or implied terms of a potential anatomical gift are in conflict with regard to the administration of measures necessary to ensure the medical suitability of a part for transplantation or therapy, the prospective donor's attending physician and prospective donor shall confer to resolve the conflict. If the prospective donor is incapable of resolving the conflict, an agent acting under the prospective donor's declaration or directive, or, if none or the agent is not reasonably available, another person authorized by law other than this [Act] to make health-care decisions on behalf of the prospective donor, shall act for the donor to resolve the conflict. The conflict must be resolved as expeditiously as possible. Information relevant to the resolution of the conflict may be obtained from the appropriate procurement organization and any other person authorized to make an anatomical gift for the prospective donor under Section 9. Before resolution of the conflict, measures necessary to ensure the medical suitability of the part may not be withheld or withdrawn from the prospective donor if withholding or withdrawing the measures is not contraindicated by appropriate end-of-life care."

## Competing interests

There are no affiliations or financial involvement with any organization or entity with a direct financial interest in the subject matter or materials discussed in the manuscript. The authors declare that they have no competing interests.

## Authors' contributions

The JLV, MYR and JLM attest they have made substantial contributions in drafting the manuscript and revising it critically for important intellectual content; that they have given final approval of the version to be published; and that they have participated sufficiently in the work to take public responsibility for appropriate portions of the content. JLV, MYR and JLM read and approved the final manuscript
